# Antioxidant-mediated up-regulation of OGG1 via NRF2 induction is associated with inhibition of oxidative DNA damage in estrogen-induced breast cancer

**DOI:** 10.1186/1471-2407-13-253

**Published:** 2013-05-22

**Authors:** Bhupendra Singh, Anwesha Chatterjee, Amruta M Ronghe, Nimee K Bhat, Hari K Bhat

**Affiliations:** 1 Division of Pharmacology and Toxicology, School of Pharmacy, University of Missouri-Kansas City, 2464 Charlotte Street, Room 5251, Kansas City, MO 64108, USA

**Keywords:** OGG1, Estrogen, Antioxidant, Oxidative stress, DNA damage, Breast cancer

## Abstract

**Background:**

Estrogen metabolism-mediated oxidative stress is suggested to play an important role in estrogen-induced breast carcinogenesis. We have earlier demonstrated that antioxidants, vitamin C (Vit C) and butylated hydroxyanisole (BHA) inhibit 17β-estradiol (E2)-mediated oxidative stress and oxidative DNA damage, and breast carcinogenesis in female August Copenhagen Irish (ACI) rats. The objective of the present study was to characterize the mechanism by which above antioxidants prevent DNA damage during breast carcinogenesis.

**Methods:**

Female ACI rats were treated with E2; Vit C; Vit C + E2; BHA; and BHA + E2 for up to 240 days. mRNA and protein levels of a DNA repair enzyme 8-Oxoguanine DNA glycosylase (OGG1) and a transcription factor NRF2 were quantified in the mammary and mammary tumor tissues of rats after treatment with E2 and compared with that of rats treated with antioxidants either alone or in combination with E2.

**Results:**

The expression of OGG1 was suppressed in mammary tissues and in mammary tumors of rats treated with E2. Expression of NRF2 was also significantly suppressed in E2-treated mammary tissues and in mammary tumors. Vitamin C or BHA treatment prevented E2-mediated decrease in OGG1 and NRF2 levels in the mammary tissues. Chromatin immunoprecipitation analysis confirmed that antioxidant-mediated induction of *OGG1* was through increased direct binding of NRF2 to the promoter region of *OGG1*. Studies using silencer RNA confirmed the role of OGG1 in inhibition of oxidative DNA damage.

**Conclusions:**

Our studies suggest that antioxidants Vit C and BHA provide protection against oxidative DNA damage and E2-induced mammary carcinogenesis, at least in part, through NRF2-mediated induction of OGG1.

## Background

Long-term estrogen use has been associated with the initiation and development of breast cancer [1-7]. The mechanisms of E2-induced breast carcinogenesis are however not clearly understood. In E2-induced breast carcinogenesis, oxidative stress produced by redox cycling between catechol estrogens and estrogen quinones is implicated to play an important role [8,9]. 8-Hydroxydeoxyguanosine (8-OHdG) is one of the most commonly formed DNA lesions produced in response to E2-induced oxidative stress and is considered as a cellular marker for both oxidative stress and oxidative DNA damage [3-5]. 8-Hydroxydeoxyguanosine in DNA is repaired primarily via the DNA base excision repair pathway. 8-Oxoguanine DNA glycosylase is the rate-limiting enzyme involved in the removal of 8-OHdG from DNA [10,11]. Association of decreased levels of OGG1 with tumor development and/or progression has been well established [12,13]. We have earlier reported that two known prototypic antioxidants Vit C and BHA can inhibit E2-mediated breast cancer development in female ACI rats [2,5,7]. The female ACI rat model is a relevant model system for human breast cancer as it shares many pertinent histopathologic and molecular features with human sporadic breast cancers, both in early pre-malignant lesions, as well as in primary tumors [14-19]. The tumors that develop in this animal model are estrogen dependent, aneuploid and exhibit genomic instability [14-19].

Protective mechanisms of action of antioxidants are often ascribed to their ability to act as free radical scavengers through induction of transcription factor nuclear factor erythroid 2-related factor 2 (NRF2)-dependent antioxidant enzymes and/or “phase II” metabolic enzymes involved in E2-metabolism [5,20,21]. NRF2 is a known regulator of the antioxidant response [22-25]. NRF2-regulated phase II enzymes protect against the development of cancer by catalyzing reactions that convert highly reactive, carcinogenic chemicals to less reactive products [22-24,26]. We have recently demonstrated that Vit C and BHA provide protection against E2-mediated oxidative DNA damage but the mechanism is not well understood [5]. In order to find a putative mechanism for inhibition of 8-OHdG formation by antioxidants Vit C and BHA, we have examined the involvement of OGG1 and NRF2. The human *OGG1* promoter contains a putative NRF2 binding site and NRF2 leads to *OGG1* transcriptional activation [27,28]. In this study, we present evidence that antioxidants, Vit C- and BHA-mediated induction of NRF2 regulates OGG1 which is involved in the inhibition of E2-induced oxidative DNA damage and possibly breast carcinogenesis in the rat model of breast cancer.

## Methods

### Treatment of animals

Female ACI rats (4 weeks of age; Harlan Sprague Dawley, Indianapolis, IN) were housed under controlled temperature, humidity, and lighting conditions. After a one-week acclimatization period, rats were divided into following different groups: Control, E2, BHA, BHA + E2, Vit C and Vit C + E2. Rats were implanted subcutaneously with 3 mg E2 pellets. E2 pellets were prepared in 17 mg cholesterol as a binder as described previously [29,30]. Control, Vit C and BHA groups received 17 mg cholesterol pellet only. Vitamin C (1%) was administered in drinking water. BHA (0.7%) was fed to animals through phytoestrogen-free AIN76A diet (Dyets, Bethlehem, PA). Water was given *ad libitum* to all the animals. Each of the six treatment groups were divided into two subgroups, containing at least 10 rats in each subgroup. Each subgroup underwent treatments as described above for 7 and 240 days, respectively. At the end of the experimental time period, animals were anesthetized using isoflurane and euthanized. Mammary tumors, mammary, liver, lung, kidney, spleen and uterine tissues were removed and snap frozen in liquid nitrogen for future analyses. The animals were treated and handled according to the guidelines of the University Animal Care and Use Committee. Animal protocols used in the current study were approved by the Institutional Animal Care and Use Committee.

### Cell culture

Non-tumorigenic human breast epithelial cell line, MCF-10A and tumorigenic human breast epithelial cell line, T47D were obtained from American Type Culture Collection (ATCC, Manassas, VA). Cells were grown in DMEM/F12 (50:50) medium (Mediatech, Herndon, VA). Twenty-four hours before treatment, cells were washed twice with PBS and then grown in phenol red-free DMEM/F12 (50:50) medium supplemented with charcoal-dextran stripped serum. Cells were treated with E2 (10 and 50 nM), Vit C (250 μM and 1 mM), BHA (250 μM), Vit C + E2, and BHA + E2 for up to 48 h.

### Real-time PCR analysis

Total RNA was isolated from ACI rat tissues and cell lines using RNeasy lipid tissue kit (Qiagen, Valencia, CA) and Tri reagent (Molecular Research Center, Inc., Cincinnati, OH), respectively, according to the supplier’s protocols. Five microgram total RNA was reverse transcribed using the superscript II reverse transcription system (Invitrogen, Carlsbad, CA). Real-time PCR was performed using iCycler iQ5 system (Bio-Rad Laboratories, Hercules, CA). Rat and human specific *NRF2* QuantiTect primers (Cat # QT00183617 and QT00027384, respectively), and rat specific *OGG1* QuantiTect primers (Cat # QT00186641) used in this study were obtained from Qiagen (Valencia, CA). Human *OGG1* specific primers used in this study were as follows: forward primer 5′-GTGCCCGTTACGTGAGTGCCAGTGC-3′ and reverse primer 5′-AGAGAAGTGGGGAATGGAGGGGAAGGTG-3′. Data were analyzed from at least 5 different animals/cell line samples from each group. The expression of cyclophilin, a housekeeping gene, was used for quantification of the mRNA levels of genes of interest [31].

### RNA interference

Small interfering RNAs (siRNAs) for *NRF2*, *OGG1* and scrambled siRNA were obtained from Santa Cruz Biotechnology (Santa Cruz, CA). MCF-10A cells were transfected with siNRF2 (20 nmol/L) or siOGG1 (5 nmol/L) using Lipofectamine 2000 transfection reagent (Invitrogen) for 48 h. Scrambled siRNA (20 nmol/L) transfected MCF-10A cells were used as negative controls as described recently [5]. MCF-10A cells transfected with siNRF2 and siOGG1 were used for western blot and DNA 8-OHdG analyses, respectively.

### Western blot analysis

Approximately 50 mg of different female ACI rat tissues were homogenized in a tissue protein extraction buffer (T-PER, Thermo Scientific, Rockford, IL). Lysates from cell lines were prepared in RIPA buffer containing a protease inhibitor cocktail (Sigma-Aldrich, St. Louis, MO). The Pierce BCA Protein Assay kit was used to determine protein concentrations (Pierce, Rockford, IL). Eighty microgram total protein from ACI rat tissues or 30 μg protein from cell lines was size fractionated on a 12% SDS-polyacrylamide gel, and transferred onto a PVDF membrane (Millipore Corp., Billerica, MA) under standard conditions [4,5,31]. OGG1 (Cat # sc-33181) and NRF2 (Cat # sc-30915) primary antibodies (Santa Cruz Biotechnology, Santa Cruz, CA) were used for immunodetection. Chemiluminescent detection was performed using the BM Chemiluminescence Detection kit (Roche, Indianapolis, IN) and Alpha Innotech FluorChem HD2 (Alpha Innotech, San Leandro, CA) gel documentation system. Membranes were reprobed with α-Tubulin antibody (Santa Cruz Biotechnology) using the methods described above. Intensities of the bands were quantified and normalized using AlphaEase FC StandAlone software (version 6.0.0.14; Alpha Innotech).

### Chromatin immunoprecipitation (ChIP) assay

Chromatin immunoprecipitation assays were performed with MCF-10A cells using ChIP Assay Kit (USB Corporation, Cleveland, OH) as suggested by the manufacturer. Briefly, MCF-10A (~5 ×10^8^) cells grown in 100 mm tissue culture dishes were treated with E2 (10 nM), Vit C (1 mM) or BHA (250 μM) for 45 min and cross-linked with 1% formaldehyde and then sonicated. Soluble chromatin was collected and incubated on a rotating platform with goat polyclonal antibody against NRF2 (Cat # sc-30915, Santa Cruz Biotechnology), overnight at 4°C. The DNA was recovered and subjected to real-time PCR analysis using primers flanking antioxidant responsive element (ARE) region of the human *OGG1* gene promoter. The *OGG1* ARE primers used for the end point real-time PCR amplification using SYBR green method (Qiagen, Valencia, CA) were as follows: forward primer 5′-GAGAACCCAGAAGAACACAG-3′ and reverse primer 5′-GTGCTGTTTAACAACCTTCC-3′. Amplification of input chromatin before immunoprecipitation at a dilution of 1:50 was used as a positive control. ChIP without any antibody served as a negative control. The assays were carried out three times with three replicates in each experiment. Agarose gel electrophoresis and Ct (cycle threshold) values for the amplified products for ChIP DNA and input DNA samples were used to represent the results.

### 8-OHdG estimation

8-Hydroxydeoxyguanosine (8-OHdG), an accepted marker of oxidative stress-mediated DNA damage, was estimated in control mammary tissues, E2-treated mammary and mammary tumor tissues as well as in E2-treated, siOGG1- or scrambled-transfected MCF-10A cells using Oxiselect Oxidative DNA Damage ELISA kit (Cell Biolabs, San Diego, CA) as described previously [4,5].

### Statistical analyses

Statistical analyses were performed by using Sigma Plot 11.0 (Systat Software, San Jose, CA) and IBM SPSS Statistics 19 software (IBM Inc., Armonk, NY). The unpaired t-test analysis was used to calculate p values for comparisons of OGG1 and NRF2 mRNA and protein levels, and 8-OHdG levels, between treated animals and respective age-matched controls as well as for comparisons in MCF-10A cells. Fisher’s exact test was used to compare tumor incidence between two treatment groups. A p value <0.05 was considered significant.

## Results

### Estrogen treatment inhibits OGG1 expression

**Figure 1:**
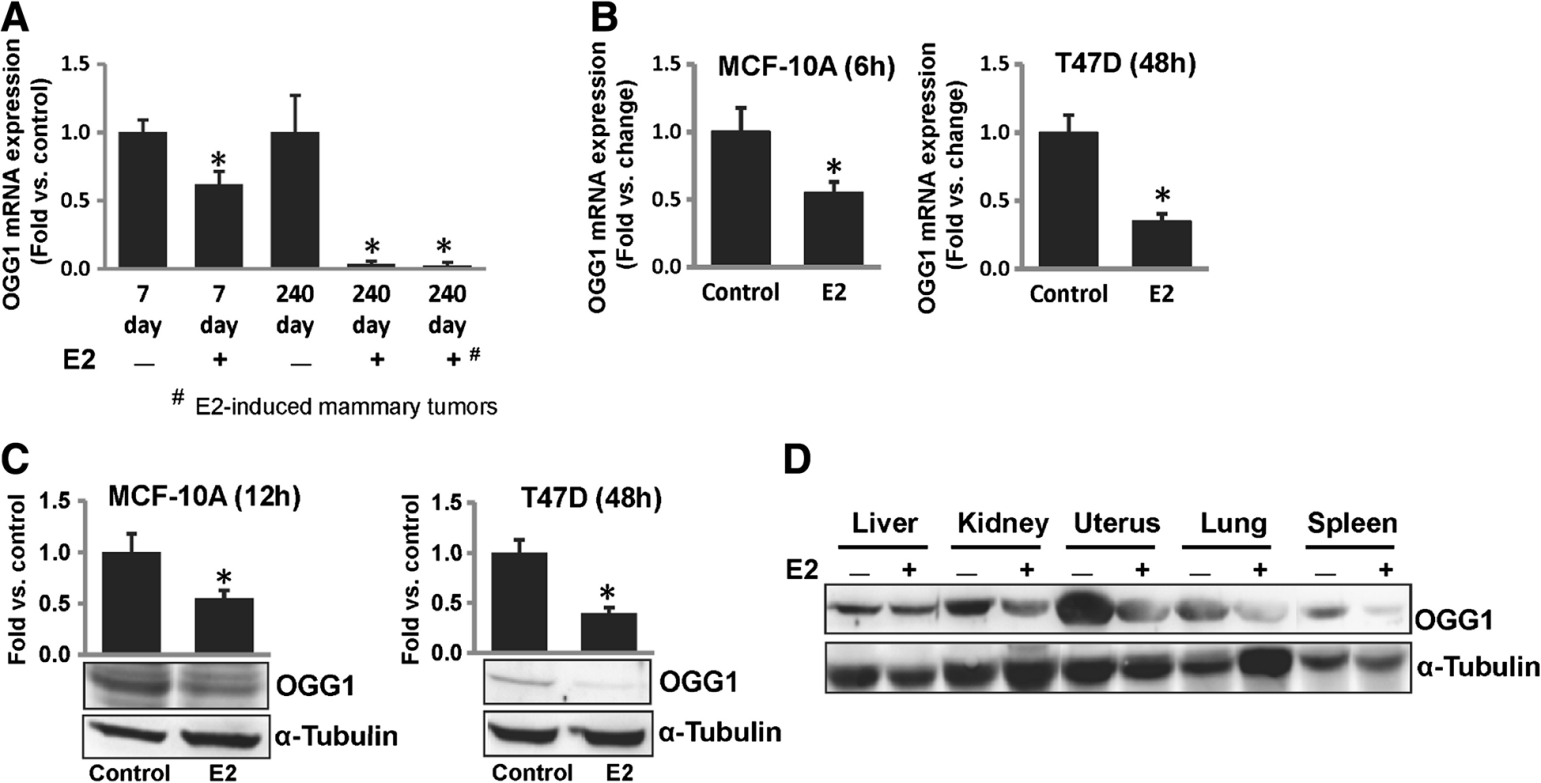
**Estrogen treatment inhibits OGG1 expression.** (**A**) Real-time PCR data are presented to show expression *of OGG1* mRNA in mammary tumors and in mammary tissues of rats treated with E2 for 7 and 240 days. These data are reported as an average of fold change values obtained from at least 5 different animals ± SEM, compared to respective age-matched control mammary tissues. (**B**) *OGG1* mRNA expression levels in MCF-10A and T47D cells treated with E2 (50 nM) for 6 and 48 h, respectively, presented as fold change versus time-matched vehicle-treated cells. *OGG1* mRNA expression data was normalized to cyclophilin as an internal control. These data are reported as an average of values obtained from at least 5 different samples ± SEM. (**C**) Representative western blots for OGG1 protein expression in MCF-10A and T47D cells treated with E2 (50 nM) for 12 and 48 h, respectively. The bar graphs represent fold change in OGG1 protein expression (mean ± SEM), compared to time-matched vehicle-treated cells. ‘*’ indicates p value <0.05 compared to respective age- or time-matched control samples. (**D**) Representative western blot for OGG1 protein expression in different tissues of female ACI rats treated with E2 for 240 days as described in “Methods” section.

We investigated the effect of E2 treatment on the mRNA expression of *OGG1* during early exposure time to estrogen (7 days) and during neoplastic (240 days) stages of breast cancer development in female ACI rats. Significant inhibition of *OGG1* mRNA expression by E2 was demonstrated in mammary tissues of rats treated with E2 for 7 days and *OGG1* mRNA expression further decreased in mammary tissues as well as in mammary tumors of rats treated with E2 for 240 days, compared to age-matched mammary tissues from control animals (Figure 1A).

We also examined *OGG1* mRNA expression *in vitro* in non-neoplastic human breast epithelial cell line, MCF-10A and in neoplastic human breast epithelial cell line, T47D. A significant decrease in *OGG1* mRNA levels in MCF-10A cells after 6 h of E2 (50 nM) treatment compared to time-matched vehicle-treated MCF-10A cells was demonstrated (Figure 1B). In contrast to MCF-10A cells, *OGG1* mRNA expression in T47D cells significantly decreased after 48 h of E2 treatment (Figure 1B). E2-mediated decrease in OGG1 protein expression was also examined in MCF-10A and T47D cells by western blot analyses. Estrogen (50 nM) treatment significantly decreased OGG1 protein expression compared to vehicle-treatment in MCF-10A and T47D cells after 12 and 48 h of treatment, respectively (Figure 1C). We observed a similar inhibitory effect of lower dose of E2 (10 nM) on OGG1 expression *in vitro* during our dose-curve analysis (data not shown).

**Figure 2:**
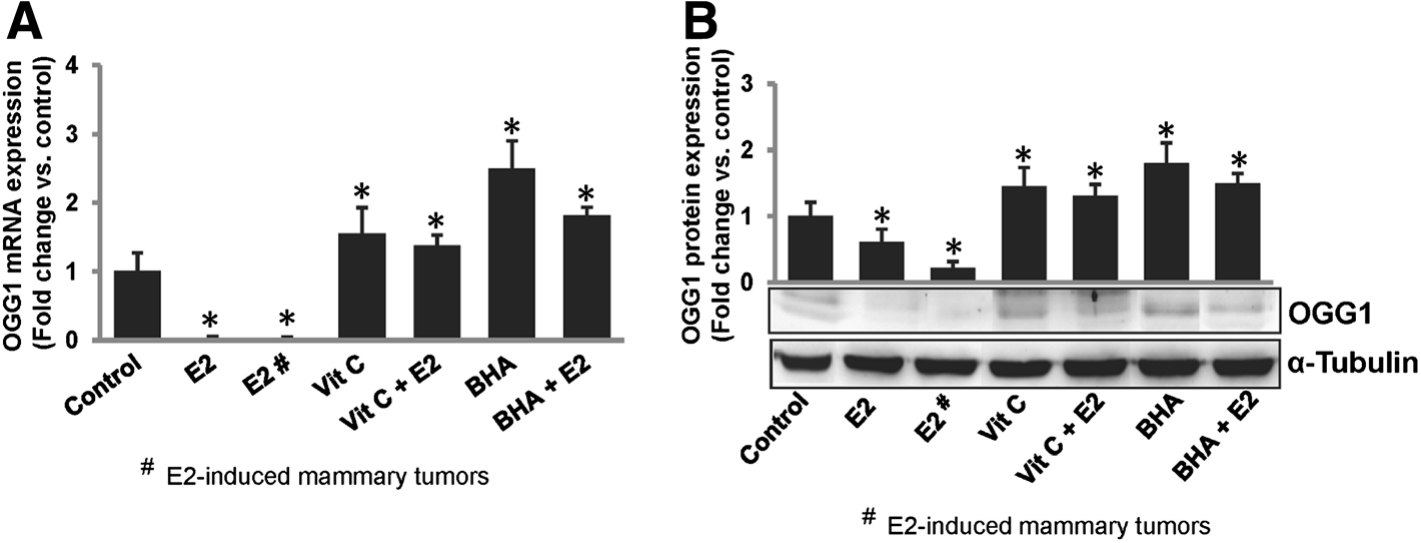
**Antioxidants Vit C and BHA prevent estrogen-mediated inhibition of OGG1.** (**A**) Real-time PCR data are presented as a bar graph to show expression levels of *OGG1* mRNA in mammary tumors and mammary tissues of rats treated with E2, Vit C or BHA in presence or absence of E2 for 240 days as described in “Methods” section. *OGG1* mRNA expression data were normalized to cyclophilin as an internal control. These data are reported as an average of values obtained from at least 5 different animals ± SEM, compared to age-matched control mammary tissues. (**B**) Female ACI rats were treated with E2, Vit C or BHA in presence or absence of E2 for 240 days as described in “Methods” section. At the end of the experiment, mammary tissues and mammary tumors were collected, homogenized and used for OGG1 western blot analysis. The bar graph represents OGG1 protein fold change (mean ± SEM) in mammary tumors and mammary tissues from at least 5 different animals from each treatment group compared to age-matched control mammary tissues. ‘*’ indicates p value <0.05 compared to age-matched control mammary tissues.

To examine whether E2-mediated suppression of OGG1 was tissue specific, we performed western blot analyses with protein samples from liver, kidney, uterus, lung, spleen, breast and breast tumor tissues from female ACI rats treated with E2 for 240 days. Estrogen treatment inhibited protein expression of OGG1 in all the tissues tested compared to age-matched respective tissues from control animals (Figures 1D and 2B).

### Antioxidants inhibit estrogen-mediated suppression of OGG1

We have recently reported that antioxidants Vit C and BHA inhibit E2-mediated oxidative stress and breast carcinogenesis in female ACI rats after 240 days of treatment [2,7]. To examine whether antioxidants Vit C and BHA also protect against E2-mediated suppression of OGG1, we performed real-time PCR and western blot analysis with mammary tissues and mammary tumor samples from rats treated with E2, Vit C or BHA in presence or absence of E2 for 240 days. While E2 significantly decreased *OGG1* mRNA and protein expression in E2-treated mammary and mammary tumor tissues compared to age-matched mammary tissues from control animals; Vit C or BHA alone or in combination with E2 protected against E2-mediated decrease in OGG1 and induced its expression at mRNA as well as protein levels (Figure 2).

### Antioxidants-mediated regulation of OGG1 is NRF2-dependent

**Figure 3:**
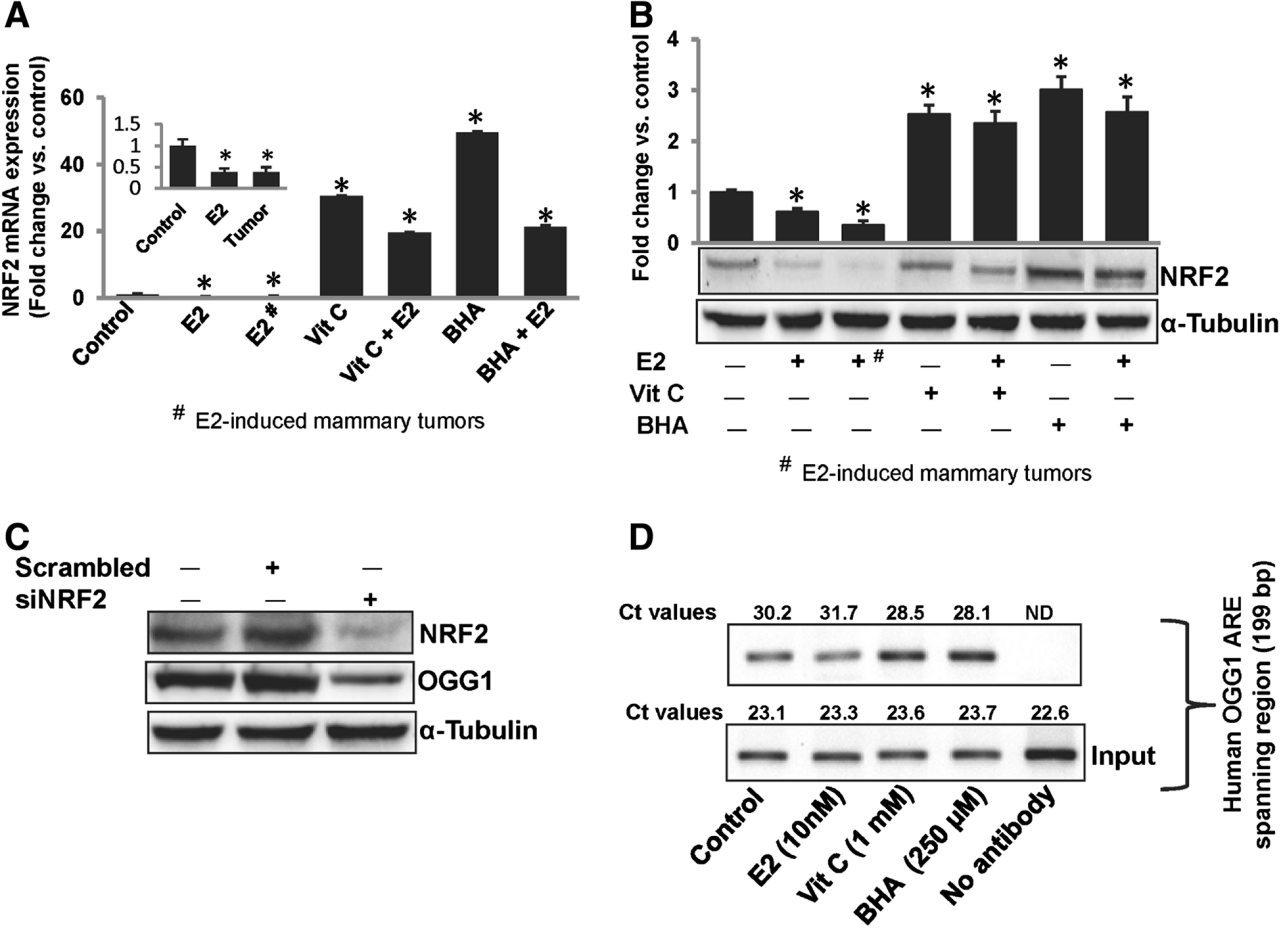
**Prevention of estrogen-mediated suppression of OGG1 by antioxidants is NRF2-dependent.** (**A**) Real-time PCR data (normalized to internal control cyclophilin) are presented as a bar graph to show expression levels of *NRF2* mRNA in mammary tumors and mammary tissues of rats treated with E2, Vit C or BHA in presence or absence of E2 for 240 days. These data are reported as an average of fold change values obtained from at least 5 different animals ± SEM, compared to age-matched control mammary tissues. ‘*’ indicates p value <0.05 compared to respective control mammary tissues. (**B**) Western blot analyses were carried out to determine protein expression levels of NRF2 in mammary tumors and mammary tissues of rats treated with E2, Vit C, Vit C + E2, BHA and BHA + E2 for 240 days. The bar graph represents NRF2 protein fold change (mean ± SEM) in mammary tumors and mammary tissues from at least 5 different animals compared to age-matched control mammary tissues. ‘*’ indicates p value <0.05 compared to respective, age-matched control mammary tissues. (**C**) MCF-10A cells were transfected with 20 nmol/L of scrambled siRNA or siNRF2 for 48 h and western blot analysis was carried out using NRF2 antibody. Same membrane was reprobed with OGG1 and α-Tubulin antibodies. Concomitant with a decrease in NRF2 protein expression, there is a corresponding decrease in OGG1 protein expression. (**D**) MCF-10A cells were treated with E2 (10 nM), Vit C (1 mM), BHA (250 μM) or vehicle for 45 min, fixed with formaldehyde, cross-linked, and the chromatin sheared. The chromatin was immunoprecipitated with NRF2 antibody. NRF2 binding to *OGG1* promoter was analyzed by real-time PCR with specific primers for the human *OGG1* ARE region. The ARE region of the *OGG1* promoter was also amplified from purified soluble chromatin before immunoprecipitation to show input DNA. Representative ChIP agarose gels and real-time PCR Ct values from three independent experiments are shown. (ND = not detected).

Transcription factor NRF2 regulates genes containing antioxidant responsive elements (ARE) in their promoter regions [5,26,32]. Presence of a putative NRF2 binding site in the human *OGG1* promoter has been reported [27,28] and presence of NRF2 binding site in rat *OGG1* promoter region has also been predicted (http://www.sabiosciences.com/chipqpcrsearch.php). We examined whether expression of OGG1 during E2-induced carcinogenesis is regulated through NRF2. We have earlier shown significant suppression of NRF2 protein expression in E2-treated mammary and mammary tumor tissues after 240 days of treatment compared to age-matched control mammary tissues [5,33]. NRF2 mRNA and protein expression was significantly increased in mammary tissues of rats treated with Vit C or BHA for 240 days, either alone or in the presence of E2 compared to age-matched mammary tissues from control animals (Figure 3A and 3B).

In parallel with decrease in NRF2 protein expression (Figure 3B), a significant decrease in OGG1 protein expression in E2-treated mammary tissues and in mammary tumors, and an increase in OGG1 protein expression in mammary tissues of animals treated with Vit C- and BHA was demonstrated (Figure 2B). A decrease in OGG1 protein expression after silencing of *NRF2* in MCF-10A cells was demonstrated which indicates NRF2-dependent regulation of *OGG1* (Figure 3C). To further confirm whether suppression of *OGG1* after E2 treatment and its induction after antioxidant treatment was through differential binding of NRF2 to the ARE present in the promoter region of *OGG1*, we carried out ChIP assay using MCF-10A cells. Following chromatin immunoprecipitation using anti-NRF2 antibody, DNA was recovered and subjected to real-time PCR analysis using PCR primers flanking the ARE region of the human *OGG1* gene promoter. Estrogen treatment inhibited the binding of NRF2 to the *OGG1* gene promoter as shown by increase in Ct values, whereas antioxidants Vit C and BHA enhanced NRF2 binding to the *OGG1* promoter as shown by decrease in Ct values compared to control (Figure 3D).

### OGG1 inhibits estrogen-induced oxidative DNA damage

**Figure 4:**
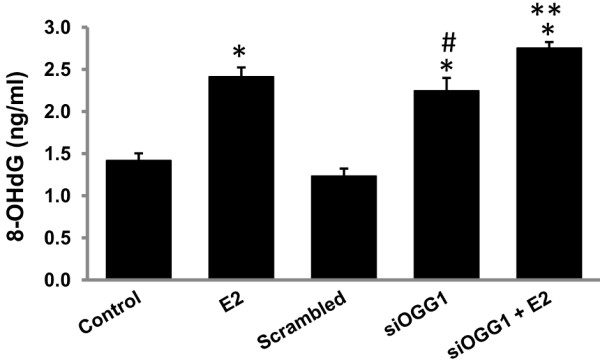
**OGG1 inhibits oxidative DNA damage.** DNA 8-OHdG levels in MCF-10A cells treated with E2 (50 nM) or vehicle for 48 h and in *OGG1* knocked down MCF-10A cells are represented as a bar graph. ‘*’ and ‘#’ indicate p value <0.05 compared to vehicle-treated control and scrambled-transfected MCF-10A cells, respectively. ‘**’ indicates significant difference (p <0.05) between siOGG1 and siOGG1 + E2 groups.

8-Oxoguanine DNA glycosylase is the main enzyme involved in the removal of 8-OHdG from DNA, and thus suggested to be involved in protection against DNA damage and subsequent carcinogenesis [10-13]. We have earlier reported that antioxidants Vit C and BHA prevent E2-mediated oxidative DNA damage and breast carcinogenesis (Table 1, [5]). Here, we demonstrate that antioxidants prevent E2-mediated suppression of OGG1 (Figure 2 and Table 1). To further examine whether OGG1 confers protection against DNA damage, we quantified DNA 8-OHdG levels in MCF-10A cell line after siRNA-mediated silencing of *OGG1*. Estrogen treatment (50 nM, 48 h) of MCF-10A cells significantly increased 8-OHdG levels compared to vehicle-treated controls (Figure 4). Similarly, a significant increase in 8-OHdG levels in siOGG1 transfected MCF-10A cells compared to scrambled siRNA transfected MCF-10A cells was observed (Figure 4). Moreover, 8-OHdG levels further increased in siOGG1-trasfected MCF-10A cells treated with E2 compared to 8-OHdG levels in siOGG1-transfected cells in the absence of E2 treatment (Figure 4).

**Table 1:** OGG1 protein expression, 8-OHdG levels and tumor incidence in female ACI rats after different treatments

**Group/ treatment**	**n**	**OGG1 protein expression (fold change vs. control)**	**8-OHdG levels (fold change vs. control)**	**Tumor incidence (%)**
Control	10	1.00	1.00	0
E2	11	0.63*	1.79*	82
Tumor	11	0.21*	2.98*	-
BHA	17	1.80*	0.89	0
BHA + E2	17	1.50*	1.23^**#**^	24^**#**^
Vit C	17	1.45*	0.95	0
Vit C + E2	17	1.31*	1.02^**#**^	29^**#**^

OGG1 protein expression and DNA 8-OHdG levels are presented in mammary tumors and in mammary tissues from control, E2, BHA, BHA + E2, Vit C and Vit C + E2-treated rats after 240 days of treatment. Column one lists different treatments each group of animals received. The number of animals per group (n) is listed in column two. Column three shows OGG1 protein expression as an average of values obtained from at least 5 different animals. Column four shows folds change in DNA 8-OHdG levels compared to age-matched control mammary tissues. The percent tumor incidence in each treatment group after 240 days of treatment is listed in column five. ‘*’ indicates a p value <0.05 compared to age-matched control tissues and ‘**#**’ indicates p value <0.05 compared to E2-treated group.

## Discussion

Prolonged exposure to elevated levels of estrogen has been implicated in the development of breast cancer [29,34,35]. Estrogens are known to induce cell growth and loss of DNA repair capacity of the cells [4,19,36]. Accurate DNA repair is essential for the prevention of mutations and ultimately, cancer. A number of recent studies have demonstrated that 8-OHdG, likely induced by estrogen-metabolism-mediated oxidative stress, is formed after estrogen exposure and suggested that 8-OHdG may be associated with estrogen-induced breast carcinogenesis [3-5]. The 8-OHdG adduct is mainly repaired by base excision repair (BER) mechanism of the cell [37,38]. 8-Oxoguanine DNA glycosylase (OGG1), an enzyme of the BER pathway, is highly specific for the removal of 8-OHdG adducts from all regions of the genome [38]. 8-Oxoguanine DNA glycosylase first hydrolyzes the glycosidic bond of 8-OHdG, then cleaves the phosphodiester bond leaving an AP site, which is repaired by DNA polymerase [37,38]. A role of this enzyme in cancer prevention/progression has been documented [12,13,39].

We have earlier shown that E2 induces oxidative stress and oxidative DNA damage during breast cancer development [1,2,5,7]. Female, ovary-intact ACI rat that we have used in our study is an established animal model for estrogen-induced breast cancer [1,17-19]. It has been reported that the serum E2 levels in control, ovary-intact ACI rats oscillate between ~20 and 75 pg/ml whereas the mean level of serum E2 in E2-treated, ovary intact female ACI rats averaged ~100 pg/ml and remained constant during the course of the study [16]. We have started E2-treatment of female ACI rats when they were 5–6 weeks old. This age is considered as puberty or early puberty stage for female rats and the duration of our study was up to 8 months which represents the fertile period of female rats. It has also been reported that female ACI rats remained in proestrus stage for the duration of study after E2 implantation [18]. We have also reported for the first time that antioxidants Vit C and BHA can prevent E2-induced oxidative stress, oxidative DNA damage and breast cancer in female ACI rats [2,5,7]. However, very little information is available about the effects of E2 and antioxidants on DNA damage repair capacity of the cells during E2-induced breast carcinogenesis. 8-Oxoguanine DNA glycosylase is a key gene responsible for repair of oxidative DNA damage [10,11]. Therefore, in the present study, we examined whether antioxidants Vit C and BHA inhibit oxidative DNA damage through regulation of *OGG1*. We have shown that E2 treatment significantly decreased *OGG1* mRNA and protein expressions in the mammary tissues (Figures 1 and 2). The decrease in *OGG1* mRNA expression in mammary tissues was evident as early as 7 days of E2 treatment and remained significantly decreased in both mammary tissues and E2-induced mammary tumors after 240 days of E2-treatment (neoplastic stage) (Figure 2A). We have demonstrated that long-term continuous E2 exposure (240 days) significantly suppressed the expression of OGG1, an enzyme involved in oxidative DNA damage repair and thus may lead to increased DNA damage in mammary tumors and mammary tissues (Figures 1A, 2, and Table 1). In our previous report, we have shown that exposure to E2 as early as 7 days can initiate proliferative changes in the mammary tissues, a progression from normal mammary tissue to proliferative tissue such as atypical ductal hyperplasia, later progressing to tumor formation and malignancy [1]. Previous studies also support E2-mediated differential expression of OGG1 in different tissues of rat [40]. Increased cell proliferation, and decreased OGG1 and thus, compromised DNA damage repair potential after 7 days of E2 treatment might be the initial steps that lead to the accumulation of carcinogenic insults at later time points.

Inhibition of OGG1 protein expression in other tissues of rats like liver, kidney, uterus, lung and spleen indicates that E2-mediated inhibition of OGG1 was not tissue specific (Figure 1D). We have earlier shown that E2 induces oxidative stress during breast carcinogenesis [1,2,7] and redox regulation of OGG1 has also been established [41]. Thus, E2-induced oxidative stress might be one of the possible mechanism of regulation of OGG1 during breast carcinogenesis. Recently, Singh *et al.* have shown that estrogen decreases the DNA repair capacity in breast cancer cells, at least in part, through epigenetic mechanism [36].

Dietary supplementation of antioxidants is suggested to reduce breast cancer most likely through induction of antioxidant enzymes and/or “phase II” metabolic enzymes but the effects of antioxidants on DNA repair capacity of the cells are not well understood [5,20,21]. In a previous study, Collins et al. found lower DNA damage in a human study population after consumption of kiwifruit given as an antioxidant supplement in the diet, but they could not find any change in expression of DNA repair-related genes *OGG1* and AP endonuclease 1 (APE1) [42]. We have earlier reported that antioxidants Vit C and BHA can prevent E2-induced oxidative stress, oxidative DNA damage and breast cancer in female ACI rats [2,5,7,33]. Significantly decreased expression of OGG1 at both mRNA and protein levels after long term E2 treatment and reversal of this suppression by Vit C and BHA in our study clearly indicates an important role of OGG1 in antioxidant-mediated protection against oxidative DNA damage as well as breast cancer (Figure 2 and Table 1). A decreased OGG1 enzyme expression level has been associated with an aggressive breast cancer phenotype [43]. To the best of our knowledge, ours is the first report showing the regulation of DNA damage repair gene *OGG1* by dietary antioxidants Vit C and BHA.

The promoter region of the *OGG1* gene does not have any canonical ERE and there is no evidence for a direct regulation of *OGG1* expression by E2. However, human OGG1 promoter contains a putative NRF2 binding site and NRF2 leads to *OGG1* transcription [27,28]. Transcription factor NRF2 is a known regulator of the antioxidant response [22-25]. We have recently shown that antioxidants Vit C and BHA upregulate expression of NRF2-regulated protective genes NAD(P)H-quinone oxidoreductase 1 (NQO1) and superoxide dismutase 3 (SOD3) in mammary tissues [5,33]. Therefore in this study, we examined whether the regulation of *OGG1* in E2-induced breast cancer is mediated through transcription factor NRF2. We have demonstrated that *OGG1* is regulated via an NRF2-dependent pathway (Figure 3). Decreased mRNA and protein expression of NRF2 and OGG1 in E2-treated mammary tissues and in E2-induced mammary tumors after 240 days of E2 treatment and corresponding increased mRNA and protein expression of these genes after Vit C or BHA treatment suggest NRF2-mediated regulation of *OGG1* (Figures 2, 3A and 3B). Decreased protein expression of OGG1 in *NRF2* knocked down MCF-10A cells confirmed NRF2-mediated regulation of *OGG1* (Figure 3C). Results from ChIP assay with MCF-10A cells treated with E2, Vit C or BHA further confirmed E2-mediated decreased and antioxidant-mediated increased binding of NRF2 to the ARE region of *OGG1* promoter and indicate a gene-nutrient interactions (Figure 3D). These results also suggest that E2-mediated oxidative stress may be involved in the regulation of OGG1. Collectively, our results provide evidence for NRF2-mediated regulation of *OGG1* in E2-induced breast carcinogenesis.

Significant increase in 8-OHdG levels (p < 0.05) in *OGG1* knocked down MCF-10A cells compared to vehicle or scrambled siRNA transfected MCF-10A cells confirmed the role of OGG1 in prevention of estrogen-induced oxidative DNA damage (Figure 4). Following E2 treatment, further significant increase in 8-OHdG levels in siOGG1-transfected cells compared to siOGG1-transfected cells without E2 treatment confirmed that the increase in 8-OHdG levels was specific to E2-induced oxidative damage (Figure 4).

## Conclusions

In conclusion, data presented in this manuscript demonstrate that antioxidants Vit C and BHA not only act as inducers of the antioxidant enzyme system but also act as the inducers of the DNA repair capacity. Our findings further suggest that antioxidants Vit C and BHA protect against oxidative DNA damage and E2-induced mammary carcinogenesis, at least in part, through NRF2-mediated induction of OGG1.

## Abbreviations

E2: 17β-estradiol; Vit C: Vitamin C; BHA: Butylated hydroxyanisole; ACI: August Copenhagen Irish; OGG1: 8-Oxoguanine DNA glycosylase; NRF2: Nuclear factor erythroid 2-related factor 2; 8-OHdG: 8-Hydroxydeoxyguanosine; ARE: Antioxidant responsive element; Ct: Cycle threshold; ChIP: Chromation immunoprecipitation.

## Competing interests

The authors declare that they have no competing interests.

## Authors’ contributions

BS and HKB conceived and designed the study. BS, AC and AMR performed the experiments. BS and HKB analyzed the data. AC, AMR and NKB contributed reagents/materials/analysis tools. BS wrote the paper. All the authors read and approved the final manuscript.

## Authors’ information

Current address: BS, Department of Genetics, School of Medicine, University of Alabama at Birmingham, Birmingham, AL 35294, USA.

## Pre-publication history

The pre-publication history for this paper can be accessed here:

http://www.biomedcentral.com/1471-2407/13/253/prepub
